# Autoimmune Limbic Encephalitis: A Review of Clinicoradiological Features and the Challenges of Diagnosis

**DOI:** 10.7759/cureus.17529

**Published:** 2021-08-28

**Authors:** Jack B Ding, John Dongas, Kevin Hu, Mark Ding

**Affiliations:** 1 Internal Medicine, Royal Adelaide Hospital, Adelaide, AUS; 2 Internal Medicine, University of Adelaide, Adelaide, AUS; 3 Internal Medicine, Lyell McEwin Hospital, Adelaide, AUS

**Keywords:** limbic encephalitis, neuroradiology, autoimmune encephalitis, receptor antibodies, neuroimaging

## Abstract

Limbic encephalitis is an autoimmune cause of encephalitis. In addition to the usual symptoms of encephalitis such as altered consciousness, fever, and focal neurological deficits, limbic encephalitis can present with neuropsychiatric manifestations and seizures. Making a formal diagnosis involves a difficult and prolonged workup phase. The purpose of this review is to help readers delineate limbic encephalitis from other illnesses. This is done by presenting a spectrum of potential organic differential diagnoses and pertinent findings that distinguish them from limbic encephalitis. Instead of presenting a variety of psychiatric differential diagnoses, the authors present a review of psychiatric manifestations known to be associated with limbic encephalitis, as naturally, any psychiatric disorder could be a potential comorbid disease.

## Introduction and background

Limbic encephalitis (LE) is an autoimmune subtype of encephalitis [1]. The term LE was first used in 1968 by Corsellis and colleagues though the literature now recognizes that inflammatory damage is not confined to the limbic region but can progress to other areas of the brain [1].

Ever since limbic encephalitis (LE) was first described, the disease has proved an ongoing challenging diagnostic dilemma. Indeed, a prolonged workup of LE is seemingly the current norm, with one case series of 20 patients noting the median time from symptom onset to a formal diagnosis being four weeks, with a range from two to 104 weeks [2].

Part of this diagnostic difficulty is due to the heterogeneity of the symptoms of limbic encephalitis and the challenge of differentiating it from other conditions. A 2019 retrospective study of 50 patients diagnosed with autoimmune encephalitis revealed that two out of three patients were originally suspected of having a different condition such as a primary psychiatric illness, a neurodegenerative disease, or epilepsy [3]. In one out of three patients in which encephalitis was a leading differential, most patients were thought to have the infectious rather than the autoimmune subtype [3]. Indeed, delineating the two subtypes can be difficult, given that fever does not reliably distinguish between the two [4].

Importantly, making an early diagnosis improves outcomes. Two observational studies in 2010 and 2016 showed that the prompt identification of LE is associated with reduced seizure frequency, improved cognitive recovery, and likely also leads to improved survival rates [5]. To our knowledge, a formal qualitative analysis on earlier detection of LE and survival rates has not been conducted. However, in the largest published series of 50 patients, it was determined that LE preceded a cancer diagnosis in 60% of cases by three and half months on average [6]. This is significant from a therapeutic perspective, given that an earlier diagnosis of LE would trigger malignancy screening and cancer treatment, which will likely yield a superior prognosis compared to delayed detection.

In this review, the authors seek to present an overview of the diagnostic tree of encephalitis, commencing with definitions for encephalopathy, followed by the characteristic features of encephalitis and its specific subtypes.

## Review

Overview of pathophysiology and subtypes

Pathophysiology of Limbic Encephalitis

**Figure 1:**
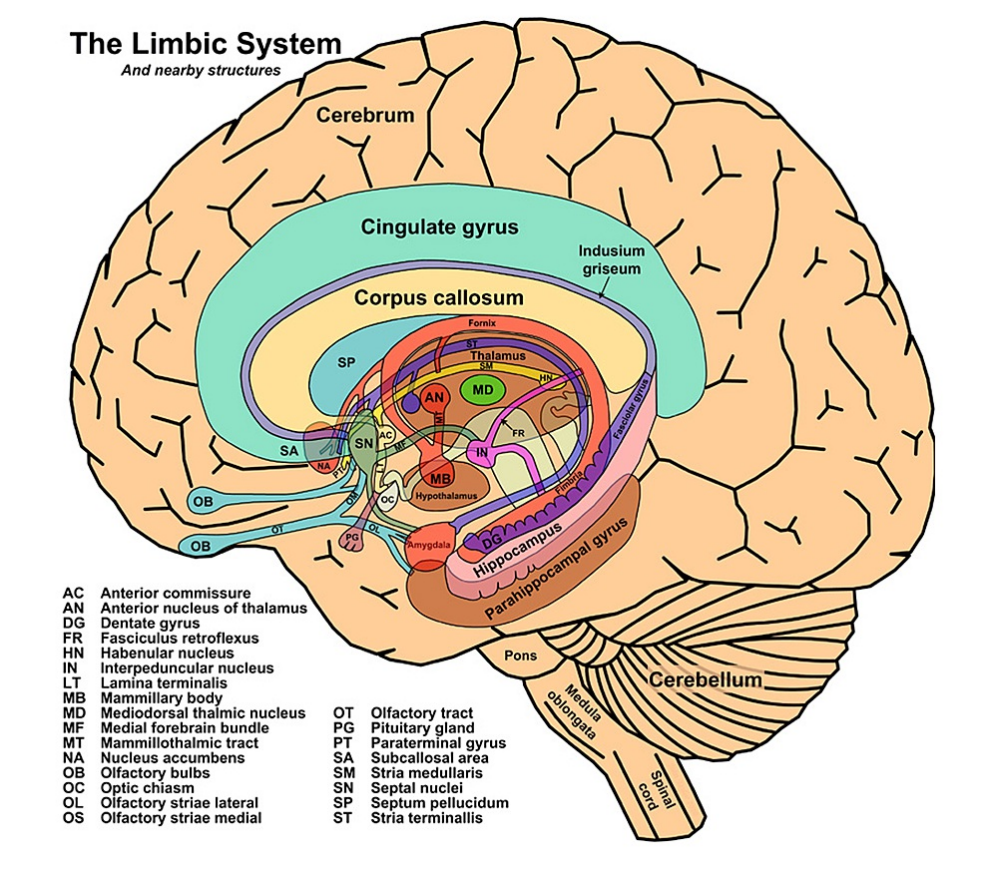
Diagram of the limbic system and its nearby structures Limbic encephalitis results in inflammation of the cingulate cortex, frontobasal cortex, hippocampus, and medial temporal lobe (image in the public domain). Source: [9]

The pathophysiology of LE is thought to be mediated by an antigen that stimulates an antibody-mediated host immune response that inadvertently targets self-antigens in the limbic area [7]. LE inflammation involves the limbic system, demonstrated in Figure 1, which includes the cingulate cortex, frontobasal cortex, hippocampus, and medial temporal lobe [8].

Damage to distinct limbic structures can cause specific neuropsychiatric manifestations, such as psychosis, disruptions to the circadian rhythm, memory lapses, mood alterations, cognitive impairment, and seizures [1]. Specifically, injury to the hypothalamus can elevate or suppress appetite and libido, damage to the amygdala can heighten aggression and emotion, and destruction of the hippocampus can result in memory and concentration deficits [10].

Although many LE cases are linked to a neoplasm, cases also occur without cancer [11]. Thus, LE is not a distinct disorder but rather an umbrella term that describes an autoimmune response that affects the limbic area [12]. The epidemiology of limbic encephalitis associated with tumors is like that of the causative malignancy. Conversely, the typical patient profile of non-tumour associated LE is a young female [13].

Since most cases of autoimmune encephalitis demonstrate a high serum to cerebrospinal fluid (CSF) antibody ratio, its autoantibodies are theorized to be of peripheral origin that subsequently penetrate the blood-brain-barrier (BBB) [14]. In terms of pathogenesis, LE antibodies target: cell-surface receptors and antigens, such as voltage-gated potassium channels and the N-methyl-D-aspartate receptors (NDMAR); LGI1 and contactin-associated protein-like 2 (CASPR2) proteins; intracellular proteins, such as Hu, Ma2, CV2, amphiphysin, and Ri; and enzymes involved in neurotransmitter metabolism [15].

Local pathologic effects following antibody-antigen interaction include direct blockade of cell-surface channels and receptors and internalization of cell-surface receptors. The autoantibodies may also activate natural killer cells or the complement system and thus promote complement-mediated or cell-mediated cytotoxicity and subsequent neuronal death [16].

Sub-categorization of limbic encephalitis is based on the specific autoantibody involved. Antibodies specific to intracellular antigens are referred to as ‘onconeural’ antibodies due to their strong association with malignancy [17]. Previously, LE has been described as a paraneoplastic syndrome involving antibodies targeting intracellular antigens. However, recent LE literature shows antibodies targeting extracellular antigen are less likely to be associated with tumors. Some examples of the autoantibodies seen, and their associated tumor type/frequency are depicted in Table 1.

**Table 1:** Limbic encephalitis antibodies, their frequency, and associated cancers (Table adapted from Budhram et al., 2019 [27]) LGI1 = leucine-rich, glioma inactivated, CASPR2 = contactin-associated protein-like 2, NMDA-R = N-methyl-D-aspartate Receptor, GABA-Br = Gamma-amino butyric acid receptor, type B, mGluR5 = metabotropic glutamate receptor 5, AMPAR = α-amino-3-hydroxy-5-methyl-4-isoxazolepropionic acid receptor, GAD = glutamic acid decarboxylase, CRMP5 = collapsin response-mediator protein-5, SCLC = small cell lung cancer

Antibody	Tumor Frequency (%)	Associated Cancer
Antibodies to extracellular antigen (cell surface or synaptic antigens)		
LGI1 [5]	10	Non-specific (thymomas, breast, and others)
CASPR2 [18]	20	Thymoma
NMDA-R [19]	40	Ovarian teratoma
GABA-Br [2]	50	SCLC
mGluR5 [20]	50	Hodgkin’s lymphoma
AMPAR [21]	60	SCLC, thymoma
Antibodies to intracellular antigen		
GAD [22]	25	SCLC, thymoma
Hu [23]	90	SCLC
Ma2 [24]	90	Testicular tumor
Amphiphysin [25]	90	SCLC, breast cancer
CRMP5 [26]	90	SCLC, thymoma

The Diagnostic Workup of Limbic Encephalitis

In the literature, encephalopathy is defined as altered mental status lasting for longer than 24 hours, with irritability, lethargy, or personality or behavioral changes [4]. While encephalitis is technically a pathological term, current literature quotes various clinical parameters to be met to justify its diagnosis. Encephalitis is defined as a state of encephalopathy with evidence of CNS inflammation, manifested by at least two of: fever; seizures or focal neurological findings attributable to the brain parenchyma; CSF pleocytosis; EEG findings suggestive of encephalitis or radiological findings suggestive of encephalitis [4].

The workup of encephalitis, therefore, involves first substantiating a general state of encephalopathy, then diagnosing encephalitis, and finally diagnosing its subtype.

The various causes of encephalopathy can be differentiated based on history, physical exam, and investigations. Important components of the history include the acuteness of symptom onset, symptom progression, and duration of symptoms.

It is important to correctly diagnose the underlying cause of encephalopathy, as many etiologies are reversible if corrected. The initial clinical assessment of the patient should include a comprehensive natural history to determine the true duration and course of illness. History findings pertaining to a specific etiology should be expanded upon. The context in which the encephalopathy presented is very important for guiding clinical assessment - possible background findings such as active malignancies or hepatic diseases or an immunocompromised status should be ascertained. Physical examination should establish any focal neurologic deficits or seizure activity. While an electroencephalogram (EEG) may be ordered to find evidence of diffuse cerebral dysfunction, the diagnosis of encephalopathy is ultimately on clinical grounds [28].

The authors present the differentials of encephalopathy in Table 2, categorized based on the pace of symptom onset and evolution.

**Table 2:** Diagnostic criteria for definite autoimmune encephalitis (Table adapted from Graus et al., 2016 [8]) CSF = cerebrospinal fluid, MRI = magnetic resonance imaging, EEG = electroencephalogram

All of the following	At least one of the following
Subacute onset (rapid progression of less than three months) of working memory deficits, seizures, or psychiatric symptoms suggesting the involvement of the limbic system	CSF pleocytosis (white blood cell count of more than five cells per mm^3^)
Bilateral brain abnormalities on T2-weighted fluid-attenuated inversion recovery MRI highly restricted to the medial temporal lobes	EEG with epileptic or slow-wave activity involving the temporal lobes
Reasonable exclusion of alternative causes	

Subtypes of Encephalitis

As seen in Tables 3-4, there are multiple causes [29], subtypes, and mimickers of encephalitis. Importantly, if encephalitis is being considered as a diagnosis, one must first rule out the immediately life-threatening subtypes such as HSV 1 with a lumbar puncture, which is frequently delayed in clinical practice [30]. After excluding viral encephalitis, the autoimmune subtype may be considered [31]. Ultimately, after a full workup, up to 62% of patients diagnosed with encephalitis may not reveal an underlying cause (Table 4) [4].

**Table 3:** Various causes of encephalopathy by time course (Table adapted from Erkkinen & Berkowitz, 2019 [29]) TB = tuberculosis, HIV = human immunodeficiency virus, NPH = normal pressure hydrocephalus, ICH = intracranial hemorrhage, SDH = subdural hematoma

	Infective	Neurologic	Other
Hyperacute		Vascular (ICH), Seizure, Migraine, Trauma	Metabolic/toxic, Hypertensive encephalopathy
Acute	Meningitis, Encephalitis, Systemic infection	Vascular (SDH), Inflammation (Demyelination)	Metabolic/toxic
Subacute	Mycotic, parasitic, TB, HIV complications	Vascular (SDH), Neoplastic (brain tumors), Limbic encephalitis	Metabolic/toxic
Chronic	Syphilis, HIV complications	Vascular (SDH), Degenerative, NPH	Metabolic/toxic

**Table 4:** Most common subtypes and mimickers of encephalitis (Table adapted from Ellul, 2018 [31])

Category	Cause
Organic etiologies	
Viral	HSV 1, HSV 2, Varicella zoster virus, enteroviruses, adenoviruses, measles virus, parechovirus, HIV
Autoimmune	Paraneoplastic limbic encephalitis Non-paraneoplastic limbic encephalitis
Mimickers of encephalitis	
Infective	Sepsis, bacterial meningitis, TB, opportunistic infections (cryptococcus, cytomegalovirus, toxoplasma)
Inflammatory	Vasculitides, systemic lupus erythematosus with CNS involvement, neurosarcoidosis, Behçet’s disease
Metabolic	Hepatic encephalopathy, toxins (drugs, alcohol), hypoglycaemic and hyponatremic states
Neoplastic	Primary brain tumors (especially low-grade glioma mimicking CNS inflammation), metastases
Others	Status epilepticus from other etiologies, aphasia and amnesia

Characteristics of specific autoimmune limbic encephalitis subtypes

General Features of Encephalitis

Possible manifestations of encephalitis, regardless of etiology, include fever, headache, seizures, lethargy, irritability, personality change, nuchal rigidity, focal neurology, coma, gastrointestinal symptoms, respiratory symptoms, rash, photophobia, and urinary symptoms [4]. The presence or absence of any symptom or sign either alone or in combination cannot definitively separate one subtype of encephalitis from another [4]. However, certain signs or symptoms are more strongly associated with one type of encephalitis than others. Seizure activity, especially in the afebrile patient, should prompt consideration of limbic encephalitis since this is frequently present in the NMDA receptor and voltage-gated potassium channel subtypes [32-33].

Clinical Course of Encephalitides Associated With Antibodies Against Glutamate Receptors

Glutamate is the main excitatory neurotransmitter and interacts with glutamate receptors (GluRs) [34]. Dysregulation in glutamate receptor modulation has been associated with several neurodegenerative and psychiatric disorders, such as Parkinson's disease and schizophrenia [35-36]. Glutamate receptors can be classified as ionotropic (iGluRs), or metabotropic (mGluRs) [37]. iGluRs function via glutamate-gated ion channels, whereas mGluRs are G protein-coupled receptors. Both the NMDA receptor and AMPA receptor are iGluRs [20]. Antibodies against the two receptors can lead to anti-NMDA receptor encephalitis and anti-AMPA receptor encephalitis, respectively. Conversely, antibodies against mGluRs can cause anti-metabotropic glutamate receptor encephalitis [20].

Clinical Course of Anti-NMDA Receptor Encephalitis

Anti-NMDA receptor encephalitis was first described in 2005 in a case series of four young women with psychiatric symptoms and ovarian teratomas [38]. Since then, this disorder has been extensively investigated through case series comprising hundreds of patients and is now the best-defined subtype of autoimmune encephalitis [19,33]. It often presents with prodromal symptoms such as fever, headache, and malaise, which is then followed by distinct neuropsychiatric manifestations [33,39]. Herpes encephalitis is recognized as a potential trigger, but other precipitants remain unclear [40]. Ovarian teratomas are identified in about 40% of cases [19], which makes screening with MRI, CT, and pelvic/transvaginal ultrasound important. The neuropsychiatric symptoms typically begin as agitation and confusion and can progress to psychosis, seizures, and autonomic instability that may require ICU-level management [19,41]. Recent literature has revealed that early treatment, including tumor removal if applicable, can lead to 80% of patients returning to a premorbid functional status, with only about 10% of patients relapsing within two years of treatment [19,42].

Clinical Course of Anti-metabotropic Glutamate Receptor Encephalitis (mGluRs)

Antibodies against the metabotropic glutamate receptor 5 (mGluR5) were first reported by Lancaster et al. in 2011, in the context of two young patients diagnosed with Ophelia syndrome, which is characterized by Hodgkin lymphoma and limbic encephalitis with psychosis and memory symptoms [43]. One 2018 study of 11 patients with anti-mGluR5 encephalitis showed that there is an associated tumor about 50% of the time, with neuropsychiatric symptoms predating a diagnosis of neoplasm by two to 11 months [20]. The same study showed that 91% of patients had a psychiatric and/or cognitive deficit, 64% had sleep disturbances, 55% had seizures, with two patients progressive to status epilepticus, and 45% had movement disorders such as clonus or dyskinesias. Another study showed that 37% of patients had an abnormal initial brain MRI, with cerebellar atrophy eventually being detected in 83% of patients [44]. That same study showed that 92% of patients were either eventually stabilized clinically or had significant improvement, and 8% had died [44]. In general, anti-mGLURi1 encephalitis causes a neuropsychiatric and cerebellar syndrome that can result in cerebellar atrophy over time, though that has the potential to improve with treatment.

Clinical Course of Anti-AMPAR Encephalitis

Encephalitis arising from 3-hydroxy-5methyl-4isoxazolepropionic acid receptors (AMPAR) is extremely rare, and the full spectrum of clinical symptoms is not yet fully elucidated [45]. The disorder was first recognized by Lai et al., in 2009 [46]. Since then, to our knowledge, no controlled prospective analysis relating to demographics, symptoms, or ideal therapeutics has been conducted. A 2020 systematic review of 55 patients found that lung cancer and thymomas were the most common associated malignancies and that 62% of total cases were linked to a neoplasm [47]. In addition, the most common symptoms were confusion (49%), amnesia (52%), convulsions (29%), and psychiatric manifestations (47%) [47].

Clinical Course of Anti-VGKC Receptor Encephalitis 

Antibodies involved in anti-voltage gated potassium channel-complex encephalitis (anti-VGKC encephalitis) are not specific to the condition. Instead, they are recognized to be present in several other conditions such as neuromyotonia and Morvan syndrome [48]. In one study of 114 patients positive for VGKC-antibodies, less than 10% were diagnosed with limbic encephalitis, with the remainder carrying a combination of non-autoimmune and autoimmune disorders, with higher titer levels being associated with the latter [48]. It is now recognized that anti-VGKC antibodies are not necessarily specific to the potassium channel itself. Another study of 96 patients with VGKC antibodies showed that 57% had antibodies to the protein leucine-rich glioma inactivated 1 (LGI1), 20% had antibodies to contactin-associated protein 2 (CASPR 2), and 3% were specific to the channel itself [49]. LGI1 is most specific for LE while CASPR2 is more specific to neuromyotonia [50].

The signs and symptoms of anti-VGKC receptor encephalitis tend to correspond to the antigen targeted. Patients with LGI-1 antibodies almost always have confusion and amnesia (100%) and seizures (92%), with hyponatremia (59%), and movement and sleep disorders being other common manifestations [49]. One study of 29 patients with LGI-1 antibodies discovered a characteristic seizure activity, described as brief, dystonic, which affected the arm and ipsilateral face - a phenomenon they termed faciobrachial dystonic seizures (FBDS) [51]. They found that 77% of patients experienced this characteristic seizure prior to the onset of confusion and amnesia that characterize anti-VGKC receptor encephalitis [51]. This seizure activity has been further investigated, with one 2018 study of patients with FBDS seizures showing that seizures were terminated with antiepileptic drugs alone in 10% of patients but 51% of patients treated with early immunotherapy recovered within 30 days of initiation [52]. Consequently, early recognition of these seizures is helpful from both a diagnostic and therapeutic perspective.

Patients with CASPR2 antibody-related encephalitis have a well-defined spectrum of symptoms, which mainly pertain to the CNS or peripheral nervous system (PNS) [53]. Notably, the progression of symptoms in this disorder is at a slower pace, and males are more often affected compared to other subtypes of limbic encephalitis. One study of 38 patients with CASPR2 antibodies showed that the top presenting symptoms were all neurological, with the most common being cognitive disturbance (26%), seizures (24%), peripheral nerve hyperexcitability (13%), and neuropathic pain (18%) [54].

One retrospective cohort study of 1992 patients with anti-VGKC antibodies showed that cerebrocortical manifestations, such as cognitive impairment and seizures, were found in 76% of patients who only had LGII antibodies compared to 29% who only had CASPR 2 antibodies [55]. Conversely, peripheral motor hyperexcitability was a feature of 21% of patients who only had CASPR 2 antibodies versus 6.5% of patients who were positive only for LGI1 [55].

Clinical Course of Anti-GABA-B Receptor Encephalitis

Antibodies targeting the GABA-B receptor causing encephalitis were first reported by Lancaster et al. in 2010. Anti-gamma-amino butyric acid receptor (GABA-BR) encephalitis typically involves behavioral changes and refractory seizures. Around 50% of cases are associated with a neoplasm, of which 90% are small cell lung cancer (SCLC) [56]. One study of 32 patients with GABA-BR antibodies showed the most prominent symptoms to be behavioral abnormalities (97%), seizures (90%), refractory status epilepticus (42%), and rapidly progressive dementia (12%) [57]. Another study of 14 cases of anti-GABA-BR encephalitis showed the typical patient profile was middle-aged or elderly males, with symptom onset typically being sudden, sometimes preceded by non-specific flu-like symptoms [58]. Lin et al. conducted a five-year prospective study in which 28 patients with anti-GABA-B encephalitis were followed up from a mortality perspective [59]. They found that 32% of patients had died, with the median survival time being 6.5 months, with the main contributory factor being tumor progression.

Clinical Course of Anti-GAD Encephalitis

Glutamic acid decarboxylase (GAD) is an intracellular protein that converts glutamate to gamma-aminobutyric acid (GABA). There are two distinct GAD antibody isoforms - GAD65 and GAD67 [60]. GAD antibodies are most frequently found in GABAergic neurons and pancreatic islet cells, with the associated neurological syndromes, such as stiff-person syndrome, limbic encephalitis, and cerebellar ataxia, thought to arise secondary to the loss of inhibitory neurotransmission from GABA [61]. A recent review of 58 patients with anti-GAD associated encephalitis showed that the most common manifestations were seizures (97%), impaired memory (59%), cognitive impairment (40%), psychiatric symptoms, mainly related to depression or personality change (28%), and status epilepticus (24%) [62].

Clinical Course of Anti-Hu Encephalitis

Hu is an RNA-binding protein located in the nuclei of neurons and plays an important role in neural development [63]. It was first described by Graus et al. in 1985 [64], in the context of SCLC in two patients. Anti-Hu encephalitis are linked to an underlying neoplasm in over 90% of cases, with the most common malignancy being SCLC [23]. The clinical features of the paraneoplastic syndrome of anti-Hu encephalitis are highly heterogeneous. A study of 72 patients reported the most common symptoms to be linked to the sensory system (54%), motor system (45%), brain stem (31%), autonomic system (28%), cerebellar symptoms (25%), and limbic systems (22%) [65]. Two other studies found the neurological symptoms to involve more than one system in 70%-78% of patients [6,66].

Clinical Curse of Anti-Ma2 Encephalitis

Anti-Ma2 encephalitis is characterized by the involvement of the limbic system, hypothalamus, and brainstem [67]. The clinical features of anti-Ma2 encephalitis correspond to deficits of these regions, with patients presenting with daytime somnolence, narcolepsy, cataplexy, and hyperphagia [24]. This subtype is also associated with supranuclear gaze palsy, hypokinesis, and dystonia that can interfere with speech and eating [68]. This constellation of symptoms is frequently mistaken for Whipple’s disease with brain involvement, with one study revealing that 16% of patients had undergone a duodenal biopsy before being worked up for a paraneoplastic syndrome [69].

Clinical Course of Anti-CRMP5 (Anti-CV2) Encephalitis

Antibodies targeting collapsin response-mediator protein-5 (CRMP5) notably affect the cerebellum and sensorimotor systems, characteristically clinically manifesting as cerebellar ataxia, uveitis, optic neuritis, with the potential to progress to encephalomyelitis (Table 5) [26].

**Table 5:** Examples of characteristic clinical findings in LE subtypes (Table adapted from Lancaster, 2016 [70]) NMDA-R = N-methyl-D-aspartate receptor, AMPAR = α-amino-3-hydroxy-5-methyl-4-isoxazolepropionic acid receptor, GABA-Br = gamma-amino butyric acid receptor type B, LGI1 = leucine-rich, glioma inactivated, CASPR2 = contactin-associated protein-like 2, GAD = glutamic acid decarboxylase

Clinical finding	Associated Antibody Subtype
Psychotic symptoms	NMDA-R, AMPAR, GABA-BR
Movement disorders	NMDA-R
Status epilepticus	GABA-BR (more characteristics) NMDA-R (more common)
Fasciobrachial dystonic seizures	LGI-1 (precedes diagnosis)
Peripheral motor hyperexcitability	CASPR2 (more common) > LGI1
Stiff-person syndrome	GAD65
Cranial neuropathies	Ma2, Hu
Cerebellitis	GAD65

Issues with imaging for limbic encephalitis

Imaging abnormalities on T2-weighted MRI sequences are a key component of the current diagnostic criteria for limbic encephalitis. Some of the challenges it presents include the diversity of imaging findings amongst the different subtypes of autoimmune encephalitis as well as the prevalence of those findings. 

Performing MRI is often delayed in the workup of patients with neuro-psychiatric presentations. Most departments would perform CT imaging prior to MRI to rule out intracranial causes of psychiatric and behavioral presentations. There is unfortunately very little literature discussing CT in the workup of patients with these presentations. The physician must use clinical gestalt in considering the need for MRI.

The literature prevalence of MRI findings in autoimmune limbic encephalitis approximates 50% of cases regardless of the underlying subtype [13]. This presents a significant challenge given that it is a key component of the diagnostic criteria and, in fact, a requirement where antibodies are unable to be isolated. Of further concern, specific subtypes have reduced frequency of imaging findings such as anti-NMDAR encephalitis, where as little as 11% of cases have neuroimaging abnormalities on initial presentation.

**Figure 2:**
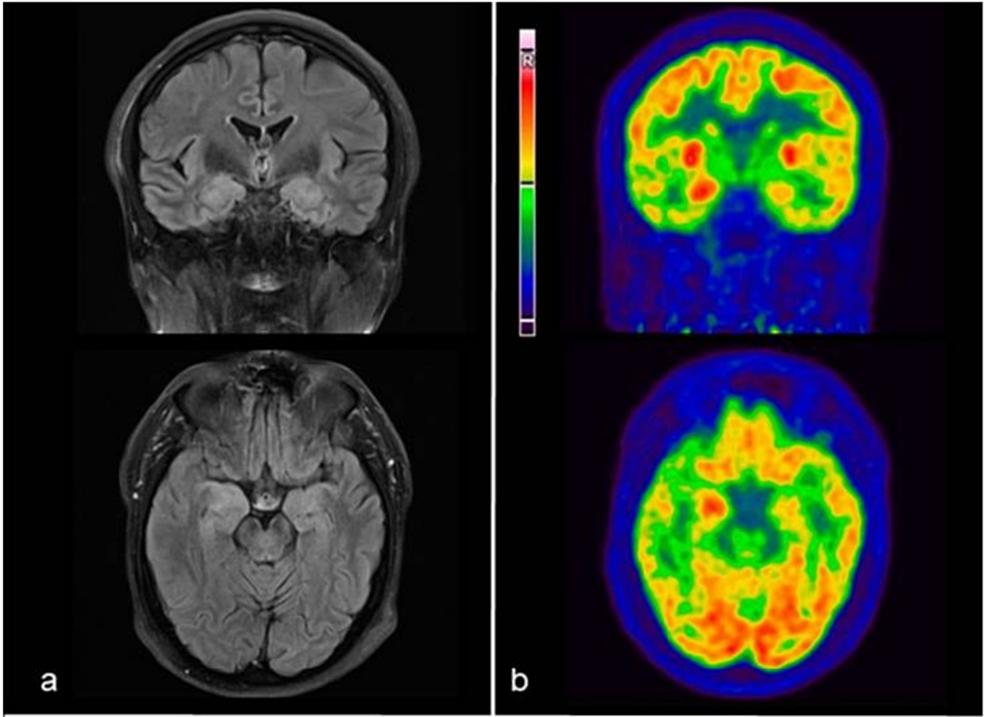
Example of anti-CASPR2 encephalitis demonstrated (a) MRI-3T, T2-FLAIR axial and coronal slices showing medial temporal hyperintensity as well as swelling, predominantly on the right side; (b) Corresponding 18F-FDG-PET/CT axial and coronal slices showing right medial temporal hyperactivity (licensed under CC BY-ND 4.0) [72] CASPR2 = contactin-associated protein-like 2, FLAIR = fluid-attenuated inversion recovery, 18F-FDG-PET = 18F-fluorodeoxyglucose-positron emission tomography

In cases where MRI is negative, there is evidence for the use of 18F-FDG PET/CT, an example of which can be seen in Figure 2. In one study, all MRI negative cases revealed abnormalities on PET/CT imaging, particularly in cases of anti-NMDAR encephalitis [71]. The main finding was not in the temporal lobes but rather of lobar hypometabolism relative to the cerebellum (most commonly parietal followed by occipital). This study was limited by its small sample size and that PET/CT was performed later than MRI. MRI however tends to remain the preferred imaging recommendation due to resources and accessibility [27].

Preliminary evidence supports the use of perfusion imaging, with the rationale being that increased perfusion may detectable before abnormalities can be seen on T2-weighted MRI imaging. This includes both CT and MR perfusion techniques, however, evidence remains limited to case reports [73-74].

## Conclusions

Autoimmune limbic encephalitis is a challenging diagnosis for several reasons. The clinical presentation can mimic various other diseases and, therefore, the differential diagnosis is large. Additionally, radiological features are frequently absent or non-specific in the more common subtypes. Being aware of the ways in which LE can present allows it to be considered as a diagnostic possibility in undifferentiated neuro-psychiatric presentations, which in turn improves treatment outcomes. Ongoing research into the pathogenesis and application of novel radiological techniques will assist in further characterizing these conditions in the future.
